# Effect of Freezing on Gut Microbiota Composition and Functionality for In Vitro Fermentation Experiments

**DOI:** 10.3390/nu13072207

**Published:** 2021-06-27

**Authors:** Sergio Pérez-Burillo, Daniel Hinojosa-Nogueira, Beatriz Navajas-Porras, Telmo Blasco, Francesco Balzerani, Alberto Lerma-Aguilera, Daniel León, Silvia Pastoriza, Iñigo Apaolaza, Francisco J. Planes, Maria Pilar Francino, José Ángel Rufián-Henares

**Affiliations:** 1 Centro de Investigación Biomédica, Departamento de Nutrición y Bromatología, Instituto de Nutrición y Tecnología de los Alimentos, Universidad de Granada, 18071 Granada, Spain; spburillo@ugr.es (S.P.-B.); dhinojosa@ugr.es (D.H.-N.); beatrizvavajas@ugr.es (B.N.-P.); spdelacueva@ugr.es (S.P.); 2 Department of Biochemistry and Molecular Biology, Boonshoft School of Medicine, Wright State University, Dayton, OH 45435, USA; 3 Tecnun, University of Navarra, Manuel de Lardizábal 13, 20018 San Sebastián, Spain; tblasco@tecnun.es (T.B.); fbalzerani@tecnun.es (F.B.); iaemparanza@tecnun.es (I.A.); fplanes@tecnun.es (F.J.P.); 4 Area de Genòmica i Salut, Fundació per al Foment de la Investigació Sanitària i Biomèdica de la Comunitat Valenciana (FISABIO-Salut Pública), 46020 València, Spain; amlermagu@gmail.com (A.L.-A.); DaLeoLop@hotmail.com (D.L.); mpfrancino@gmail.com (M.P.F.); 5 CIBER en Epidemiología y Salud Pública, 28001 Madrid, Spain; 6 Instituto de Investigación Biosanitaria ibs.GRANADA, Universidad de Granada, 18071 Granada, Spain

**Keywords:** gut microbiota, freezing, storage, foods, bioactive compounds

## Abstract

The gut microbiota has a profound effect on human health and is modulated by food and bioactive compounds. To study such interaction, in vitro batch fermentations are performed with fecal material, and some experimental designs may require that such fermentations be performed with previously frozen stools. Although it is known that freezing fecal material does not alter the composition of the microbial community in 16S rRNA gene amplicon and metagenomic sequencing studies, it is not known whether the microbial community in frozen samples could still be used for in vitro fermentations. To explore this, we undertook a pilot study in which in vitro fermentations were performed with fecal material from celiac, cow’s milk allergic, obese, or lean children that was frozen (or not) with 20% glycerol. Before fermentation, the fecal material was incubated in a nutritious medium for 6 days, with the aim of giving the microbial community time to recover from the effects of freezing. An aliquot was taken daily from the stabilization vessel and used for the in vitro batch fermentation of lentils. The microbial community structure was significantly different between fresh and frozen samples, but the variation introduced by freezing a sample was always smaller than the variation among individuals, both before and after fermentation. Moreover, the potential functionality (as determined in silico by a genome-scaled metabolic reconstruction) did not differ significantly, possibly due to functional redundancy. The most affected genus was *Bacteroides*, a fiber degrader. In conclusion, if frozen fecal material is to be used for in vitro fermentation purposes, our preliminary analyses indicate that the functionality of microbial communities can be preserved after stabilization.

## 1. Introduction

The gut microbiota is known to affect host health, playing a critical role in the modulation of the physiological processes of the related to chronic conditions such as diabetes type II [1], colorectal cancer [2], obesity [3], neurological disorders [4], inflammatory bowel disease [5], celiac disease [6], or food allergies [7]. Therefore, the possibility of optimizing the gut microbiota via diet and bioactive compounds has received much attention. However, this task has a number of challenges due to the complexity of diets, the microbiome, and human physiology, and requires different approaches ranging from in vivo to in vitro or in silico studies [8].

There is a lack of consensus in gut microbiota research when it comes to the best procedures for sample collection, sample storage, in vitro fermentation protocols, sequencing techniques, or data analysis. Regardless, it is essential to follow a proper protocol that ensures the most reliable outcome [9]. One of the key logistic steps in gut microbiome studies that has to be considered is how to store fecal material. Authors across our bibliography agree that the storage of volunteer feces has to be treated carefully since it can have a profound effect on the microbial community [10]. The effect that storage time and temperature can have on fecal microbial communities has been extensively studied in healthy subjects [10,11,12,13,14], subjects with type II diabetes [15], or with inflammatory bowel disease [16,17]. Freezing the fecal sample at −80 °C shortly after collection if prolonged time until analysis is required is considered the “gold standard” [10]. However, other storage conditions have also been studied, particularly different preserving solutions [18,19] or freeze-drying [20]. Nevertheless, for the fecal material transplant (FMT) consensus [21], the “gold standard” is to store the feces with glycerol at a 50:50 proportion at −80 °C, as glycerol will allow for the preservation of the cell structure, which is essential for FMT.

Regardless, authors agree that differences among individuals greatly surpass the effects that storage could cause on the microbial community. This is true when only the DNA in the fecal material is analyzed since the DNA is preserved in the sample. However, freezing fecal material could have an undesirable effect if those feces are to be used for in vitro fermentations, which are conducted to study how gut microbes metabolize specific bioactive compounds (i.e., phenolics or fiber) or even specific foods. Some especially sensitive bacteria could be killed during freezing or may not be able to resume their metabolic activity as fast as others, needing a longer revivification period. This could mean that some other microbes could outcompete them, which could change cross-feeding mechanisms and other ecological interactions. Therefore, the resulting microbial community could display a very different functionality from the one it initially had. However, this issue is highly understudied and very little information is currently available. Fouhy et al. [12] performed plate cultures of total aerobes, anaerobes, and bifidobacteria and did not find anysignificant differences between using fresh or −80 °C frozen feces. On the other hand, in a mucosal artificial colon in vitro model, Deschamps et al. [14] did find differences at the microbial family level depending on how feces were stored, though they only used fecal material from two healthy volunteers.

Here, within the framework of the Stance4Health European Commission project, the aim of this paper is to shed some light onto the field by studying the differences caused by using fresh or frozen fecal material for in vitro fermentation purposes, using lentils as an example food. In order to better assess whether different fermentation assay outcomes can be detected among the frozen samples of different individuals, we employed fecal material from four children, one being healthy and lean, and the other three being celiac, obese, or allergic to cow’s milk, respectively. The preliminary results of this research will help establish the effects of freezing fecal material for in vitro fermentation purposes related to the study of either specific bioactive compounds or specific foods.

## 2. Materials and Methods

### 2.1. Samples

Boiled lentils were bought at a local grocery store (Granada, Spain). The lentils were washed with bi-distilled water to remove all the preservation liquid before in vitro digestion.

### 2.2. In Vitro Gastrointestinal Digestion of Lentils

Boiled lentils were digested in vitro following the gastrointestinal digestion protocol described by Brodkorb et al. [22]. First, 5 g of boiled lentils (*n* = 10) bought at a local grocery store were ground and mixed with a salivary solution with alpha-amylase (150 U/mL) at a 50:50 *w*/*v* ratio. The mixture was kept at 37 °C for 2 min in oscillation. Next, 10 mL of gastric solution with pepsine (4000 U/mL) was added, and the pH was adjusted to 3. The mixture was kept at 37 °C for 2 h in oscillation. Finally, 20 mL of intestinal solution with pancreatine (200 U/mL) and bile salts (20 mM) were added, and the pH was adjusted to 7. The mixture was kept at 37 °C for 2 h in oscillation. Afterwards, the tubes were submerged in ice for 15 min to stop the enzymatic reactions. Tubes were centrifuged for 10 min at 4500× *g*, and the sedimented pellet was recovered. This pellet is the not digested fraction and therefore is not absorbable in the small intestine, which goes into the large intestine, becoming a substrate for gut microbes. That pellet was the substrate of in vitro fermentation.

### 2.3. Fecal Material Collection

Fecal material was collected from four children, one being healthy and lean, and the other three being celiac, obese, or allergic to cow’s milk, respectively. Children were males between 9–10 years old. Fecal material was collected using a stool collector (FECOTAINER, AT Medical BV, Oosterhout, The Netherlands). Fecal material was kept at 4 °C and transported to the laboratory in a thermal bag within 4 h. All parents provided an informed consent for fecal material collection.

Once in the laboratory, fecal material from each child was homogenized and aliquoted to be used fresh that same day or to be frozen at −80 °C. Fecal material was frozen mixed with glycerol (20%) in a 50:50 *w*/*v* proportion, following the guidelines for fecal material transplantation [21]. A fecal inoculum was prepared by mixing fecal material with pH 7 0.1 M phosphate buffer at a concentration of 32% *w*/*v*. The same procedure to obtain the fecal inoculum was followed with frozen fecal material. Manipulation of fecal material was always done under anaerobic conditions (80% N_2_, 16% CO_2_ and 4% H_2_).

### 2.4. In Vitro Fermentation

**Figure 1:**
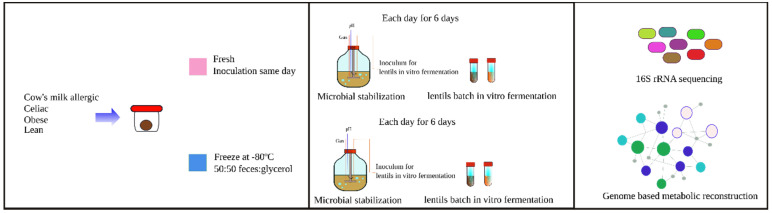
Scheme of the experiment.

In vitro fermentation consisted of two steps carried out in parallel. Microbial communities were stabilized in a complete medium for 6 days. Every 24 h, an aliquot from the stabilization medium was taken and used to inoculate a batch of the lentils used for the in vitro fermentation (Figure 1).

#### 2.4.1. Stabilization

The stabilization medium was composed of starch 4 g/L, mucin 4 g/L, yeast extract 3 g/L, arabinogalactan 1 g/L, xylan 1 g/L, peptone 1 g/L, pectin 2 g/L, glucose 0.4 g/L, cysteine 0.5 g/L, hemin 0.02 g/L, bile salts 0.5 g/L, and vitamin K1 10 ug/L [23]. Ingredients were dissolved in milli-Q water and autoclaved, except cysteine and vitamin K1, which were filtered and added afterwards.

Inoculation of the stabilization medium was performed with 100 mL of fecal inoculum into 400 mL of medium. The stabilization medium was kept at 37 °C under continuous stirring. Anaerobic conditions were reached by bubbling N_2_ and CO_2_ into the medium. The pH was kept at 7. Each day, enough volume from the stabilization medium was removed and centrifuged to produce the microbial sample for the in vitro fermentation of the lentils. The same volume of fresh stabilization medium was added to the stabilization vessel.

The experiment was carried out twice following the same procedure but using either the fresh or frozen fecal material. When the frozen fecal material was used, it was first centrifuged to remove the glycerol.

Aliquots were also taken to study gut microbial community structure. Six samples (6 days) were obtained for each fecal inoculum (celiac, obese, allergic, and lean). Therefore, 24 fresh fecal material samples were obtained and another 24 samples using frozen fecal material. Samples obtained from the stabilization medium will be referred to as STZ from here on.

#### 2.4.2. Lentils Batch Fermentation

In vitro fermentation of lentils was carried out as in [24]. First, 10 mL of stabilization medium were centrifuged, and the supernatant was removed. The pellet contained the stabilized microbial community. Next, 9.5 mL of fermentation medium (peptone + cysteine + sodium sulfide) and 0.5 g of digested lentils were added to the tube. In vitro fermentation was kept at 37 °C for 20 h in oscillation. Immediately after, the tubes were submerged in ice to stop the microbial fermentation, and the aliquots were taken and stored at −80 °C until further analysis could be completed.

Two sets of samples were analyzed, one for fresh fecal material and another for frozen fecal material, each set consisting of six samples (6 days) for each fecal inoculum (celiac, obese, allergic, and lean). Samples obtained from the in vitro fermentation of lentils will be referred to as LF from here on.

### 2.5. 16S rRNA Gene Amplicon Sequencing and Bioinformatic Analysis

DNA extraction from the solid residues deriving from the fermentation process was performed using a MagNA Pure LC JE379 platform (Roche, Basel, Switzerland) and the DNA Isolation Kit III, with an initial lysis with lysozyme at 0.1 mg/mL. An amount of 12 ng of microbial genomic DNA was used as a template for the amplification of the V3–V4 hypervariable region of the 16S rRNA gene, following the Illumina protocol for 16S Metagenomic Sequencing Library Preparation (Cod. 15044223 Rev. A). PCR primers were as described by Klindworth et al. (2013) [25], with the forward primer (5′-TCGT CGGC AGCG TCAG ATGT GTAT AAGA GACA GCCT ACGG GNGG CWGCA-G3′) and the reverse primer (5′-GTCT CGTG GGCT CGGA GATG TGTA TAAG AGAC AGGA CTAC HVGG GTAT CTAA TCC3′). Primers contained adapter sequences to make them compatible with the Illumina Nextera XT Index kit. Amplicon libraries were pooled and sequenced in an Illumina MiSeq sequencer in a 2 × 300 cycles paired-end run employing the MiSeq Reagent kit v3.

The DADA2 (v1.8.0) package as implemented in R (v3.6.0) was employed for sequence processing and the merging of forward and reverse sequencing reads as well as for the generation and annotation of amplicon sequence variants (ASVs) [26]. Filtering and trimming parameters were as follows: maxN = 0, maxEE = c(2,5), truncQ = 0, trimLeft = c(17,21), truncLen = c(270,220), and rm.phix = TRUE. A minimum overlap of 15 nucleotides and a maximum mismatch of 1 were required for read merging. Reads were aligned against the human genome (GRCh38.p11) using Bowtie2 and matches were discarded. Sequences with 100% similarity were clustered into ASVs [27]. The SILVA132 reference database (v1.12) was used for the taxonomic annotation of the ASVs [28]. Each ASV was assigned the lowest possible taxonomic rank. When attainable, species rank was assigned using the *assignSpecies* method within DADA2, which uses exact string matching against the reference database to assign Genus species binomials. Only ASVs that match a unique reference sequence at 100% identity are assigned at species level rank. Computational simulation analyses employing a large set of 16S rRNA gene sequences from finished genomes indicate that this exact matching of ASVs is an appropriate method for species-level assignment based on 16S rRNA gene hypervariable regions (Edgar 2018).

### 2.6. Functional Analysis Using Gut Microbiota Metabolic Networks

Constraint-based modeling (CBM) analysis was carried out using a recently published metabolic network of the human gut microbiota [29]. This metabolic model allows us to generate context-specific gut microbiota metabolic models with available omics data. Here, for each sample, model contextualization was performed with 16S rRNA gene amplicon sequencing data and the culture media used in the different conditions. We included those ASVs with *assignSpecies* identifications corresponding to species incorporated in [29] in the contextualization. In order not to leave other species that were highly likely to be present in our dataset out of our model, we also included ASVs with 97% identity to the species incorporated in [29], but only when no other species in the SILVA132 database matched the ASV with >95% identity. In addition, once rarefaction was applied to the smallest library size, only ASVs with a relative abundance above 0.01% of the total microbial community were considered present in each sample. After removing low abundance species from each sample, we determined the potential list of active reactions and metabolic pathways via Flux Variability Analysis (FVA) [30]. Finally, we compared the coverage of the annotated metabolic pathways between the fresh and frozen fecal samples as well as the involved gut microbiome species. All the analyses were performed using MATLAB version 2018a (The MathWorks, Inc., Natick, MA, USA).

### 2.7. Statistical Analysis

Principal coordinates analysis using Bray–Curtis dissimilarity was carried out as an exploratory multivariate analysis for ASVs whereas for metabolic reactions, Jaccard dissimilarity was used since the data were binary. Distance-based redundancy analysis (db-RDA) also using Bray–Curtis or Jaccard dissimilarity was carried out as interpretative analysis. Db-RDA tries to explain the variability found in the microbial community (or metabolic reactions data set) via different environmental explanatory variables (condition (i.e., lean, celiac, allergic or obese) and whether samples were fresh or frozen). It also explains whether these explanatory variables have a significant (*p* < 0.05) influence on the variability of the data set. Variance partitioning analysis describes how much of the variance can be explained by each of the environmental variables. Orthogonal partial least squares discriminant analysis (OPLS-DA) was carried out as a multivariate discriminant analysis. OPLS-DA is an algorithm that, after training, allows the prediction of which group the each of the samples belongs to according to specific features. In addition, it explains how important each feature is (i.e., each ASV) to classify the sample into one or another (i.e., celiac, allergic, obese, or lean; fresh or frozen) [31]. The higher the importance of the feature to classify the samples, the more differentially abundant that feature will be between the fresh and frozen samples. Model fitting is given by the parameters R2Y and Q2. The first one reflects the percentage of variation explained by the response variables, and the latter explains the proportion of variance in the data predictable by the model [32]. Finally, pair-wise comparisons were made via the Wilcoxon test (*p* < 0.05). All data analysis was carried out using R version 3.6.3.

## 3. Results

### Effect of Freezing Fecal Samples on Gut Microbiota Community Structure

This paper describes the effect of freezing fecal material as a means of preservation before in vitro fermentation experiments. Fecal material was collected from a healthy lean child, a child allergic to cow’s milk, an obese child, and a celiac child. Each stool was then divided in two fractions, one to be used fresh (on the day of collection) and the other to be frozen at −80 °C with glycerol following the guidelines for FMT [21]. The microbial community in the fecal samples was incubated over 6 days in a complex medium as in [23], with the aim of giving the cells time to recover from the effects of freezing. As a control, the fresh samples were also incubated in the same manner. Each day, aliquots were taken to analyze the microbial community composition at each time point (Figure 1). Another aliquot was taken for use as microbial inoculum in the in vitro batch fermentation of lentils. Each day, (6 days total), lentils that were in the in vitro batch were fermented with the inoculum obtained that same day from the stabilization medium. Additionally, microbial community structures obtained at each time point in either the stabilization medium or the fermented lentils were used to contextualize a human gut microbiota metabolic network (GMMR) and obtain the microbial metabolic pathways present in each sample.

**Figure 2:**
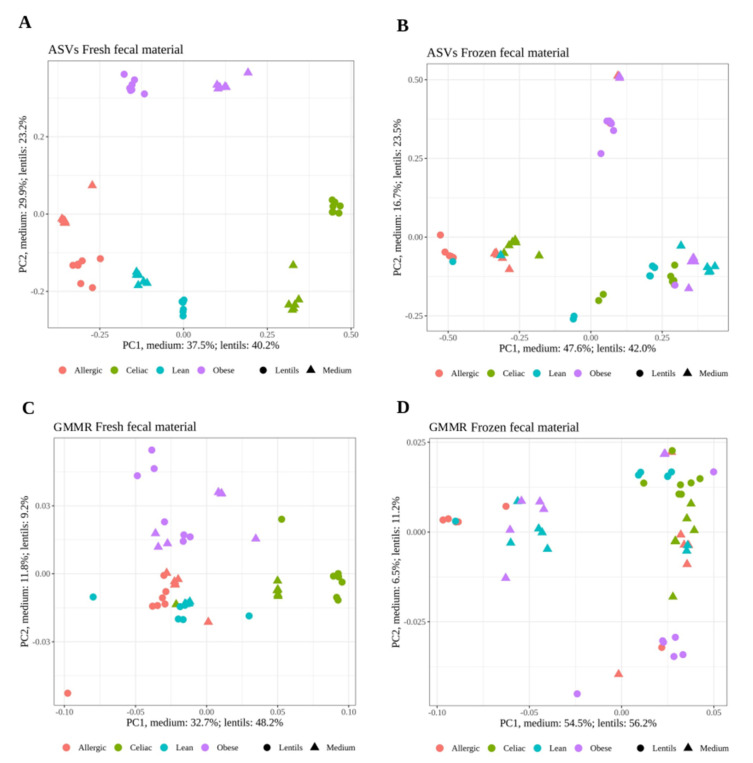
Principal coordinates analysis (PCoA) with Bray–Curtis dissimilarity of the gut microbial community structure (ASVs) for fresh fecal material (**A**) and frozen fecal material (**B**) and with Jaccard dissimilarity for active reactions after contextualizing a gut microbiota metabolic network (GMMR) of fresh fecal material (**C**) and frozen fecal material (**D**) after stabilization and after in vitro fermentation of lentils. Dots and triangles in the same color represent the samples from six different days.

First, the distribution of the stabilization medium samples and the fermentation samples were explored via Principal Coordinates Analysis (PCoA) with Bray–Curtis distance using the ASVs obtained via 16S rRNA gene amplicon sequencing as input. As depicted in Figure 2A, when fresh fecal material was used, the samples were well separated according to the donor individual (i.e., celiac, obese, allergic, or lean) in both the STZ and LF samples. This shows that the microbiota of each individual changes little during the stabilization process in relation to inter-individual variation. However, when frozen fecal material was used, the separation was not so clear (Figure 2B). The output reactions obtained after contextualizing our GMMR were also studied via PCoA with Jaccard distance (Figure 2C,D). As with the ASVs, separation according to individuals was better when using fresh fecal material than when using frozen fecal material in both the STZ and LF samples. Regardless of the fecal material used, separation among the individuals was not as evident as it had been in the case of the ASVs, which would indicate that the microbiotas of different individuals are more similar functionally than structurally. This is likely due to the ability of several different bacteria to carry out the same metabolic function, which is known as functional redundancy [33].

Additionally, dissimilarity values were studied under three situations: (i) dissimilarity between conditions, analyzing fresh and frozen samples separately (Lean vs. Celiac; Lean vs. Allergic; Lean vs. Obese; Celiac vs. Allergic; Celiac vs. Obese; Allergic vs. Obese), aimed to address whether each individual’s microbiome was still distinguishable from that of other individuals after freezing; (ii) dissimilarity between fresh and frozen samples of each condition aimed to determine whether freezing results in different microbiomes (i.e., lean microbial community using fresh fecal material vs. lean microbial community using frozen fecal material and so forth); and (iii) dissimilarity within fresh and frozen samples through time aimed to investigate whether microbiomes evolve differently after freezing (i.e., dissimilarities between different stabilization and fermentation days within the same individual).

**Figure 3:**
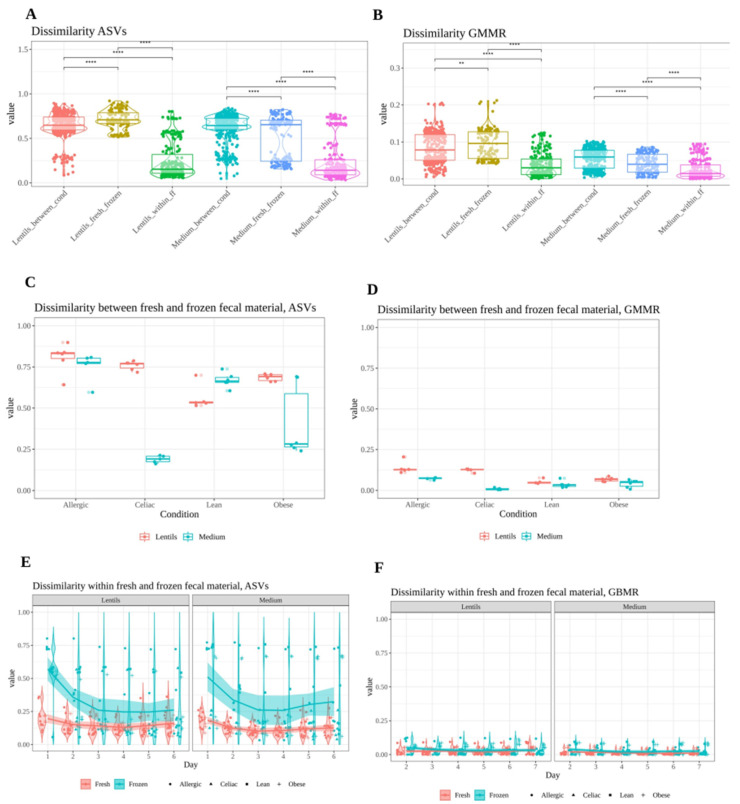
Bray–Curtis (ASVs) and Jaccard (GMMR) dissimilarity values. (**A**,**B**) show overall dissimilarity values in the three different situations tested for ASVs and GMMR for all days of the experiment considered together; (**C**,**D**) show dissimilarity values between fresh and frozen fecal material. Comparisons were made for each condition: day 1 fresh vs. day 1 frozen; day 2 fresh vs. day 2 frozen; day 3 fresh vs. day 3 frozen; day 4 fresh vs. day 4 frozen; day 5 fresh vs. day 5 frozen; day 6 fresh vs. day 6 frozen; (**E**,**F**) show dissimilarity values between stabilization and in vitro fermentation days. Here, each day is compared to the rest. Statistical labels: ** *p* < 0.01; **** *p* < 0.0001.

Overall dissimilarity values for all days of the experiment considered together are depicted in Figure 3A,B for ASVs and GMMR. As expected, dissimilarities between conditions and between fresh and frozen samples were much higher than dissimilarities within the fresh or frozen samples of the same individual over time, both significantly (*p* < 0.05). Nevertheless, dissimilarities between fresh and frozen samples were substantial. According to these results, using frozen fecal material for in vitro fermentations could result in a microbial community different to that obtained when using fresh fecal material.

ASV dissimilarity values between conditions (i.e., Lean vs. Celiac; Lean vs. Allergic; Lean vs. Obese; Celiac vs. Allergic; Celiac vs. Obese; Allergic vs. Obese) were large in both fresh and frozen samples, indicating that even after freezing, individual microbiomes could be distinguished from each other (Figure S1a). There were some exceptions, however: in the LF samples, the lean and celiac microbiomes were much more similar to each other after freezing, while, in STZ samples, celiac and allergic as well as lean and obese microbiomes were more similar to each other after freezing. GMMR’s dissimilarity values between the conditions were much lower than those of ASVs’, reinforcing the idea of functional redundancy (Figure S1b). Additionally, differences remained after freezing, showing that this treatment barely affected dissimilarity between conditions.

Secondly, dissimilarity values between fresh and frozen samples were compared within the same condition (i.e., lean microbial communities using fresh fecal material vs. lean microbial communities using frozen fecal material and so forth). Regarding microbial community structure (i.e., ASVs), large dissimilarity values were obtained for all conditions except in celiac and obese from the stabilization medium (Figure 3C). Regarding GMMR, dissimilarity values were much lower (Figure 3D). Those large ASV dissimilarity values found between fresh and frozen samples could mean a different bacterial growth patterns in the recovery medium, which ultimately could result in a different microbial community structure. Freezing fecal material could hamper the initial growth of bacteria that needed longer revivification times. If some bacteria do not grow as well as they would in fresh fecal material, this could result in a modification of the ecological interactions within the community [34]. For instance, competition for substrates could be altered and, as a result, cross-feeding metabolites, bacteria benefiting from them, etc., could also be affected [35,36]. Therefore, a few bacteria being affected by freezing could translate into a profound change in the microbial community structure. However, as stated before, dissimilarities regarding potential functionality were much lower, reinforcing again the idea of functional redundancy [37].

Dissimilarity values within conditions (with either fresh or frozen fecal material) were also studied (i.e., differences between stabilization or fermentation days within each condition and within fresh or frozen samples) in order to analyze how the community or the functionality evolved during stabilization. ASV dissimilarity values using fresh fecal material were stable during stabilization, except on day 1, when they were higher, suggesting that the community was already stabilized by day 2 (Figure 3E). When frozen fecal material was used, ASV dissimilarity values were higher than for fresh fecal material, especially on day 1, but decreased drastically by day 2 and continued to decrease by day 3, suggesting that the community took a little longer to stabilize (2–3 days). GMMR’s dissimilarity values were much lower than those of the ASVs for both fresh and frozen samples (Figure 3F).

It was then tested via distance-based redundancy analysis (db-RDA) whether the condition (celiac, allergic, obese, or lean) and freezing had a significant influence on the microbial community structure and its functionality. Regarding the ASVs, condition (*p* = 0.004975) as well as freezing the fecal material (*p* = 0.009950) played a significant role in shaping the community structure. However, regarding GMMR, while the condition played a significant role (*p* = 0.004975), freezing the fecal material did not (*p* = 0.079602). In addition, we performed a variance partitioning analysis to unravel how much of the variance was explained by either of the variables. The influence of the condition was always higher than that of freezing the fecal material in both the STZ and LF samples. The condition explained 35.6% of the variance in the STZ samples and 9.6% in the LF samples, whereas freezing explained 9.3% of the variance in the STZ samples and 2.2% in the LF samples. Results for GMMR followed the same tendency; the condition explained 45.3% of the variance in the STZ samples and 15.1% in the LF samples, whereas freezing only explained 5.0% of the variance in the STZ samples and 1.4% in the LF samples.

**Figure 4:**
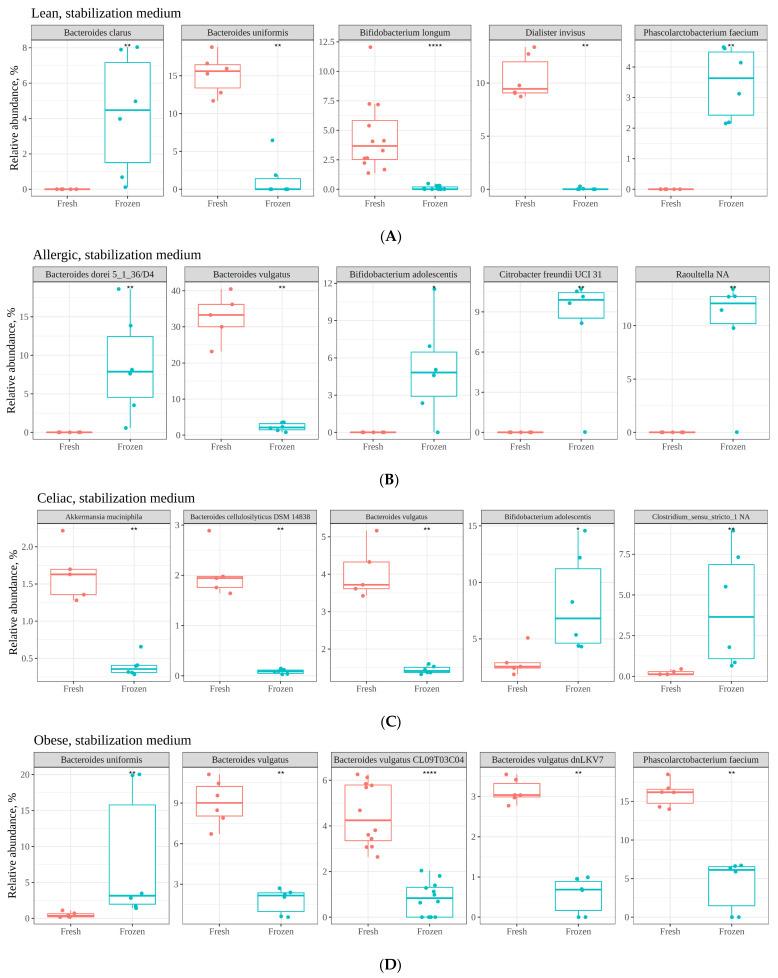
Top five discriminant ASVs between fresh and frozen samples in the stabilization medium, as determined via OPLS-DA. (**A**) show the results obtained with fecal samples of lean children. (**B**) show the results obtained with fecal samples of children with allergy to cow’s milk. (**C**) show the results obtained with fecal samples of celiac children. (**D**) show the results obtained with fecal samples of obese children. Statistical significance was obtained via Wilcoxon test (*p* < 0.05). Significance labels: ns: not significant; * *p* < 0.05; ** *p* < 0.01.

As the results have suggested so far, freezing fecal material for further use for in vitro fermentation purposes results in significantly different microbial community structures than the ones obtained if the fecal material were fresh. Therefore, the next step was to perform a discriminatory analysis to highlight which microbial members were differently abundant between fresh and frozen samples. Orthogonal partial least squares discriminant analysis (OPLS-DA) was applied to point out those discriminant ASVs. Eight models (4 for the STZ samples and 4 for the LF samples) were built: fresh lean vs. frozen lean, and so forth. Model fitting is given by the parameters R2Y and Q2: the first one reflects the percentage of variation explained by the response variables, and the latter explains the proportion of variance in the data predictable by the model [32]. Regarding the stabilization medium, fitting parameters for all four models were good: R2Y was in the range of 0.935–0.998 and Q2 was in the range of 0.740–0.988. The top five discriminant bacteria for each condition are depicted in Figure 4A–D. According to these results, the *Bacteroides* genus seems specially affected, with several ASVs (ASV0002, ASV0042, ASV0003, ASV0012, ASV0033, and ASV0035) present at a lower abundance in frozen samples. On the other hand, *Bacteroides* ASV0004 and ASV0013 were present at a lower abundance in fresh samples. It is also worth mentioning that ASV0019 (*Bifidobacterium*) was found at higher levels in the frozen samples from the celiac and allergic children, and ASV0054 (*Akkermansia*) was found at lower levels in the frozen samples from the celiac child.

**Figure 5:**
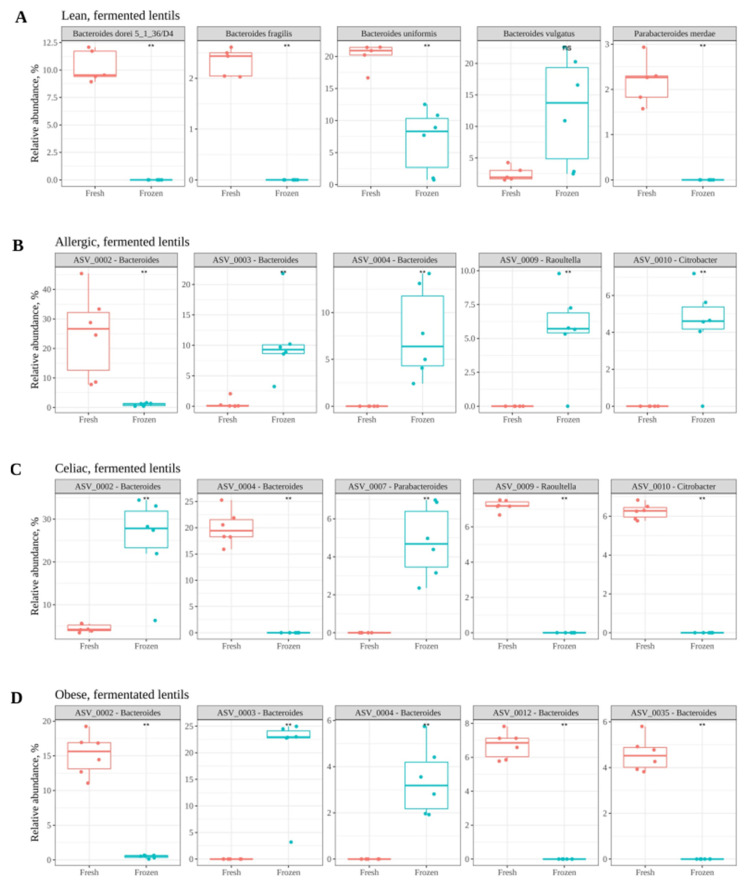
Top five discriminant ASVs between fresh and frozen samples after in vitro fermentation of lentils, as determined via OPLS-DA. (**A**) show the results obtained with fecal samples of lean children. (**B**) show the results obtained with fecal samples of children with allergy to cow’s milk. (**C**) show the results obtained with fecal samples of celiac children. (**D**) show the results obtained with fecal samples of obese children. Statistical significance was obtained via Wilcoxon test (*p* < 0.05). Significance labels: ns: not significant; ** *p* < 0.01.

Regarding the fermented lentils, the fitting parameters for all four models were good: R2Y was in the range of 0.916–0.992 and Q2 was in the range of 0.847–0.980. The top five discriminant bacteria for each condition are depicted in Figure 5A–D. As depicted in the figures, the genus *Bacteroides* was again highly affected. *Bacteroides* species are known to be major fiber degraders and have also been shown to display both competition among each other as well as cooperation and cross-feeding mechanisms releasing fiber degradation products [37].

The data presented here so far have shown how microbial communities evolve differently if they are used after being frozen. Since the initial microbial community is the same, one likely explanation is that freezing could particularly hinder the growth of some bacterial species. These bacterial species could then be outcompeted by others that normally (using fresh fecal material) would not be predominant. In addition, cross-feeding interactions could be affected and therefore, the growth of the bacteria depending on the metabolites produced by such interactions could be affected as well. Accordingly, some apparently minor initial changes in the microbial community could translate into more substantially different microbial communities after a period of growth.

**Figure 6:**
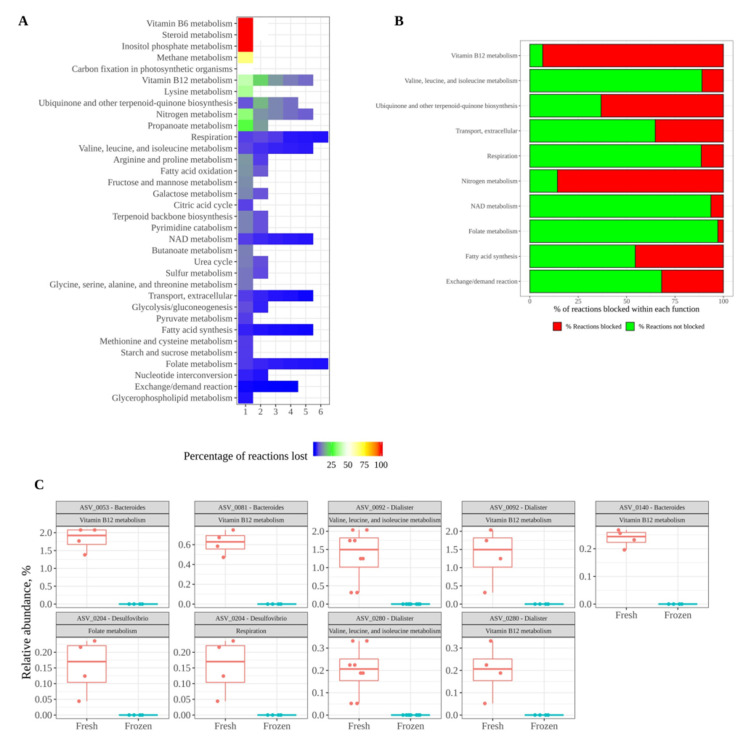
(**A**) shows a heatmap of the percentage of reactions that are present in fresh samples but not in frozen samples over the course of the stabilization days; (**B**) shows the percentage of reactions that are blocked in each function as a consequence of the reactions lost on day 5; (**C**) shows the microbes related to lost functions and how they were present in fresh samples but not in frozen samples.

Finally, the microbial metabolic pathways that were not present after freezing the fecal material were studied. As depicted in Figure 6A, on the first stabilization day, many different reactions from different metabolic functions were not present in the frozen samples. However, as shown in the same figure (Figure 6A), the number of such reactions progressively decreased during stabilization days. Nevertheless, there were some functions with missing reactions until the last two stabilization days: vitamin B12 metabolism; nitrogen metabolism; valine, leucine, and isoleucine metabolism; fatty acid synthesis; NAD metabolism; extracellular transport; respiration; and folate metabolism. Further, folate metabolism and respiration did not recover the missing reactions. These data show how microbial communities need to be stabilized for several days in order to recover their functional potential, that and even with a 6-day stabilization period, not all reactions were recovered. Moreover, the loss of some reactions could also hamper further reactions that depend on the metabolites generated by the former, thus compromising entire metabolic pathways. Figure 6B shows the percentage of reactions that are blocked in each function as a consequence of the reactions lost on day 5. Given that the metabolic reconstruction performed is based on the species present in a given sample according to 16S rRNA gene analysis, the lack of certain reactions means that all of the microbes that encode them are also missing. Figure 6C depicts the microbes related to such functions and how they were present in fresh samples but not in frozen samples. These microbes, therefore, could be specially affected by freezing and could have an impact on the functionality of the microbial community.

## 4. Discussion

In vitro fecal gut microbiota fermentations are an essential tool to study the relationship between gut microbiota, human health, and diet (both whole foods or bioactive compounds) [38,39]. However, to carry out these experiments, it is often not possible to use fresh fecal material for a number of reasons: donors are not able to provide it on time, the magnitude of the experiment does not allow it to be finished in just one day, and so forth. Therefore, the best solution is to freeze the fecal material and thaw it (not more than once) when needed. In fact, the “gold standard” is to store the fecal material right after collection at −20 °C or below [10]. In order to minimize damage to bacterial cells during freezing, several protocols and chemicals have been proposed. For fecal material transplantation (FMT), though, the gold standard is considered to be the freezing of fecal material with 20% glycerol 50:50 *w*/*v* [21]. Indeed, several studies demonstrate how after freezing the fecal matter with a given cryoprotector, the fecal microbial community barely changes, or the modifications are not significant. However, the performance of the cryoprotection has scarcely been studied for in vitro fermentation purposes. Some members of the microbial community are likely to be affected by freezing, and their growth afterwards could be impaired or delayed and thus, so is their metabolic activity. To resolve this, researchers usually stabilize the microbial community in a complex nutritive medium for bacterial revivification for at least 24 h. However, that does not change the fact that some microbes could have been more affected by freezing than others and, in the revivification medium, their growth and metabolic activity could lag behind that of their competitors and other interacting microbes. That would mean that ecological relations within the microbial community could change. For instance, a specific microbe not growing at its usual rate could mean that another, usually outcompeted by the former, grows at an unusually higher rate; cross-feeding metabolites may then change and trigger some other community-wide consequences. As a result, the microbial community obtained after revivification could be somewhat different from that before freezing.

Accordingly, a pilot experiment to test whether freezing fecal material had a significant effect on the gut microbial community for in vitro fermentation purposes was performed. The results show how the gut microbial community structure obtained after using frozen fecal material was significantly different as demonstrated by PCoA and db-RDA. However, based on the reactions predicted to be present in the bacterial communities using GMMR, the comparison of the fresh and frozen samples indicated that the potential functionality was not as affected. Moreover, the influence of the condition (i.e., obese, lean, allergic, or celiac) in both the gut microbial community structure and functionality was higher than that of freezing the fecal material. In fact, potential functionality was not significantly affected by freezing the fecal material, according to db-RDA. We performed OPLS-DA between the fresh and frozen samples within each condition to find the most discriminant microbes between these samples. In this sense, the *Bacteroides* genus was especially affected. This genus is composed of many species, and many of them are known to play major roles in fiber degradation [37,40]. At the same time, competition mechanisms [37] have been described within the *Bacteroides* genus when it comes to fiber degradation as well as cases of cooperation and cross-feeding. The data suggest that freezing could alter the growth of some microbes and in doing so, it could alter the competitive environment and thus cause further alterations in ecological relations that could distort the microbial community structure.

On the other hand, GMMR-based prediction of the reactions occurring in the microbial communities suggests that metabolic potential was much less affected by the freezing of the fecal material. One possible reason is that the functionality of a complex microbial community is much more robust than the actual structure due to functional redundancy. As shown in the results section, most of the reactions that were affected were recovered during stabilization, indicating that some bacteria need a revivification period after being frozen. However, there are some reactions that remained missing after 5–6 stabilization days. This could indicate that some bacterial species either need longer revivification periods or are unable to recover from freezing.

In conclusion, the data presented here suggest that the freezing of fecal material can result in a somewhat different gut microbial community structure, although the functionality of the community is largely maintained. Freezing fecal material would indeed impair the growth of some bacteria and as a consequence, other competitive bacteria could grow in their place, triggering a change in other ecological relations such as cross-feeding. Therefore, we recommend that the choice between using fresh or frozen fecal material should be made depending on the purpose of the study. Fresh fecal material would be preferable if researchers want to focus on how the composition of the gut microbiota behaves. Nevertheless, the fact that the variation introduced by freezing a sample is smaller than the variation existing among individuals, both before and after fermentation, indicates that fermentation experiments with frozen samples can still be informative. However, some bioactive compounds (i.e., phenolics) are metabolized by very specific species, that are also usually in low abundance [41]. Therefore, if they are lost during freezing, it will be impossible to study those specific metabolic activities. On the other hand, thanks to functional redundancy, most of the metabolic pathways can be carried out by several microbial species and, if the aim of the work is not focused on specific microbes, fecal material can be frozen. Specific undigested food residues or some dietary components (i.e., fiber, proteins, etc.) will still be used by gut bacteria, and a study with frozen fecal material can point to those microbes or features to be looked at with more specific experiments. Finally, ecological relationships within a microbial community are extremely complex and thus, these data can only suggest potential explanations, and further experiments with pure cultures and co-cultures of bacteria will be needed to confirm the effect of freezing on bacterial competition and other interactions.

## Data Availability

Supplemental data are published online with this publication.

## Supplementary Materials

The following is available online at https://www.mdpi.com/article/10.3390/nu13072207/s1, Figure S1: Panel A, Bray–Curtis dissimilarity between conditions based on ASV composition; Panel B, Jaccard dissimilarity between conditions based on GMMR.
