# Supplementary material for: Effect of Freezing on Gut Microbiota Composition and Functionality for In Vitro Fermentation Experiments
Source: Nutrients. 2021 Jun 27;13(7):2207. doi: 10.3390/nu13072207 (PMC8308218; doi:10.3390/nu13072207)

Effect of freezing on gut microbiota composition and functionality for in vitro fermentation experiments

Supplemental Information

Supplemental Figure S1. Panel A shows Bray-Curtis dissimilarity values between conditions for ASV composition. **Panel B** shows Bray-Curtis dissimilarity values between conditions for the GMMR.

A

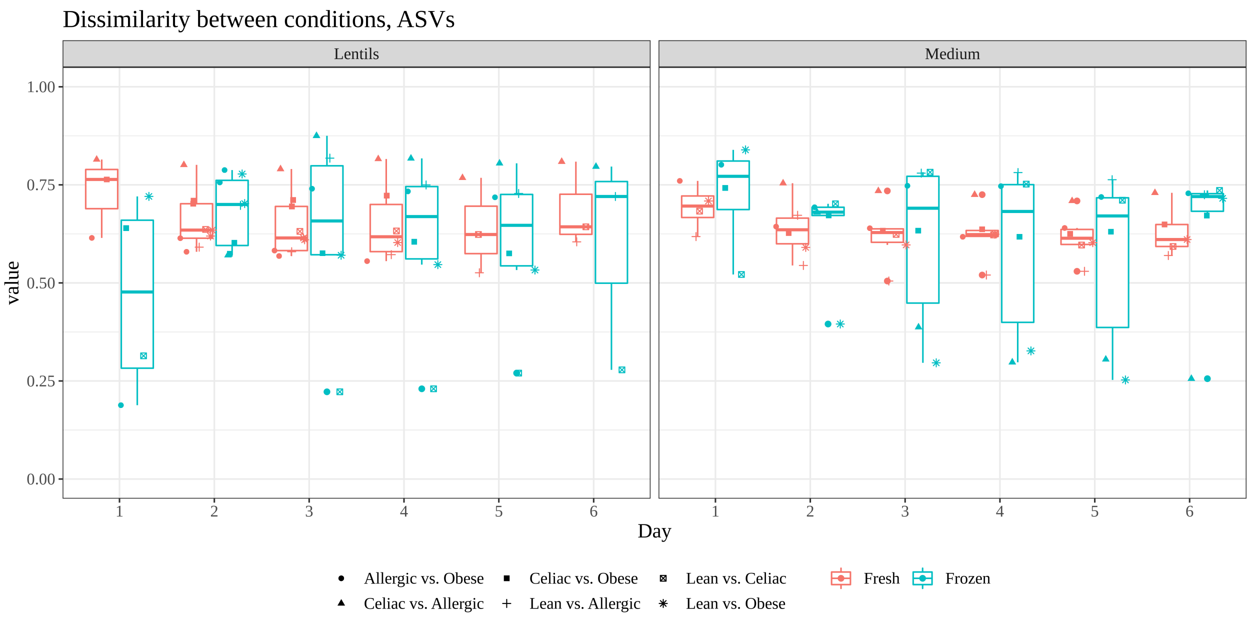

B

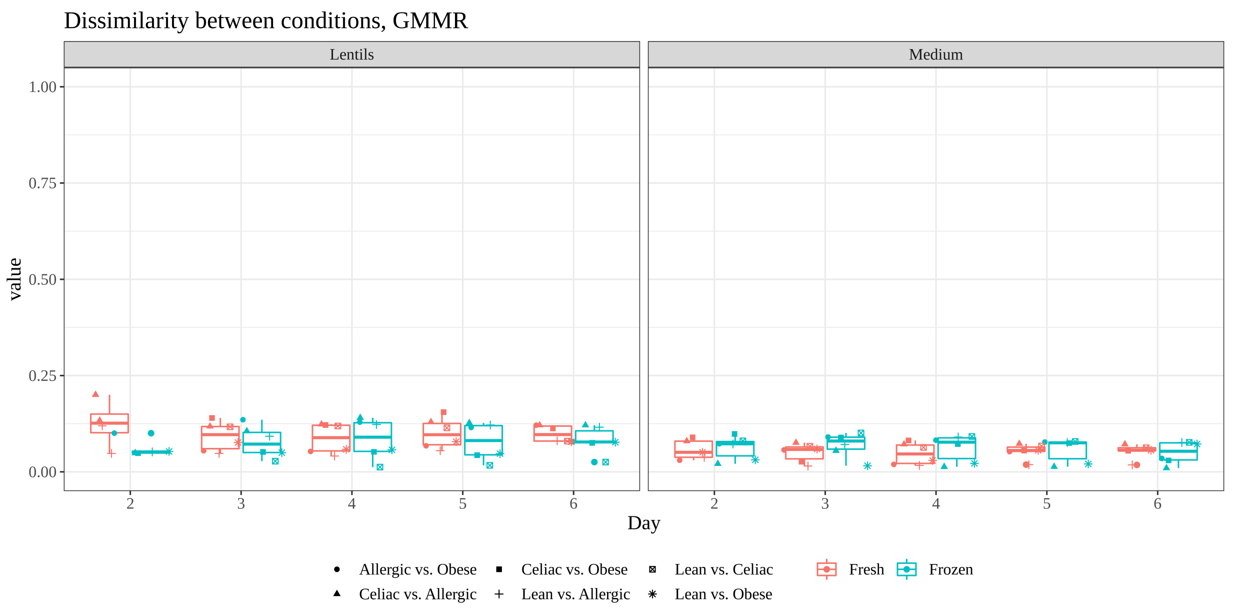

Supplement: Supplementary file 1 [file nutrients-13-02207-s001.zip › nutrients-1257488-supplementary.pdf]
